# From Learning to Memory: What Flies Can Tell Us about Intellectual Disability Treatment

**DOI:** 10.3389/fpsyt.2015.00085

**Published:** 2015-06-03

**Authors:** Alaura Androschuk, Basma Al-Jabri, Francois V. Bolduc

**Affiliations:** ^1^Department of Pediatrics, University of Alberta, Edmonton, AB, Canada; ^2^Neuroscience and Mental Health Institute, University of Alberta, Edmonton, AB, Canada

**Keywords:** intellectual disability, *Drosophila*, learning, memory, fragile X, clinical trials

## Abstract

Intellectual disability (ID), previously known as mental retardation, affects 3% of the population and remains without pharmacological treatment. ID is characterized by impaired general mental abilities associated with defects in adaptive function in which onset occurs before 18 years of age. Genetic factors are increasing and being recognized as the causes of severe ID due to increased use of genome-wide screening tools. Unfortunately drug discovery for treatment of ID has not followed the same pace as gene discovery, leaving clinicians, patients, and families without the ability to ameliorate symptoms. Despite this, several model organisms have proven valuable in developing and screening candidate drugs. One such model organism is the fruit fly *Drosophila*. First, we review the current understanding of memory in human and its model in *Drosophila*. Second, we describe key signaling pathways involved in ID and memory such as the cyclic adenosine 3′,5′-monophosphate (cAMP)–cAMP response element binding protein (CREB) pathway, the regulation of protein synthesis, the role of receptors and anchoring proteins, the role of neuronal proliferation, and finally the role of neurotransmitters. Third, we characterize the types of memory defects found in patients with ID. Finally, we discuss how important insights gained from *Drosophila* learning and memory could be translated in clinical research to lead to better treatment development.

## Introduction

Intellectual disability (ID), previously known as mental retardation, is a common neurodevelopmental disorder affecting 3% of the population and frequently a lifelong condition (1, 2). ID is defined by impaired general mental abilities and adaptive functions with an onset before the age of 18 (3). Adaptive functions are divided into three main categories: (i) conceptual, which includes reasoning, executive function, and problem solving; (ii) social, which involves interpersonal communication and relationship; and (iii) practical, which involves personal care and activities of daily living (3). Mental abilities are assessed with intelligence tests such as the Weschler Scale Intelligence in Children, which includes verbal and non-verbal components. ID has been defined with intellectual quotient (IQ) scores less than 70. ID’s severity is frequently based on IQ: Mild ID 51–70, moderate 36–49, severe 21–35 and profound <20.

**Figure 1:**
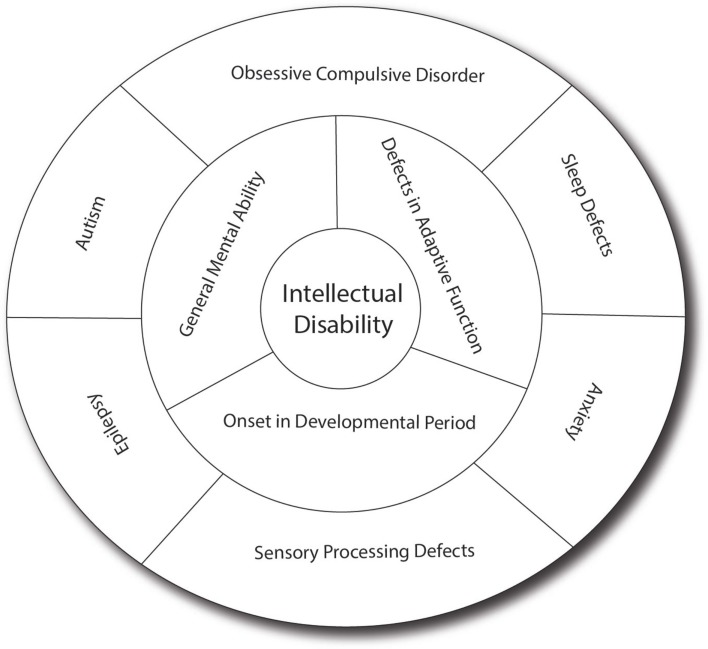
**Intellectual disability and related co-morbid conditions**. Intellectual disability is defined by the presence of cognitive defects (as measured usually by an intellectual quotient below 70) and the presence of adaptive dysfunction. These symptoms must have an onset before the patient is 18 years old to distinguish it from other conditions such as dementia. Several other conditions are frequently associated with ID, including sleep difficulties, obsessive-compulsive behaviors, anxiety, sensory-processing difficulties, autism, and epilepsy, which have significant effect on the cognitive and adaptive behaviors of the patients. In many patients, the effect of anxiety can be so prominent that it leads to underestimation of the true cognitive potential. Sensory-processing deficit is also very important in regulating the perception of sound, light and pain, and can contribute to the anxiety. Autism, which involves limited behavioral and social repertoire, will also affect the ability of the child to interact and thus impact his development. Epilepsy is much more common in ID (up to 30% as opposed to 3% in the general population) and can have detrimental effect on memory and daily functioning. In addition, drugs used to treat epilepsy (such as valproic acid) may have effects on memory themselves.

Intellectual disability can present as isolated cognitive defects or in association with other symptoms or physical signs. ID patients should be assessed for co-morbid conditions such as autism, epilepsy, anxiety, obsessive-compulsive disorder, and sleep disorders (4) (Figure 1). Physical signs can be characteristic facial or body features (known as dysmorphism) or even magnetic resonance imaging (MRI) structural abnormalities. The systematic association of specific signs has been defined as syndromic ID. Non-syndromic ID represents the other ID patients without clear dysmorphic or MRI malformations. Syndromic ID facilitates the clinical diagnosis of individuals but has also allowed for faster gene discovery and can still facilitate the interpretation of results in exome or genome sequencing by increasing the number of patients whose genome can be compared for common mutations. Traditional syndromes have been recognized and mapped to specific gene regions (Table 1). It is important to note that some syndromes are more frequent in specific populations and should therefore be screened first. For instance, a high rate of carrier for the mutation causing Tay Sachs is seen in Ashkenazi Jews and a high incidence of Bardet Biedl is found in Bedouin family. The study of ID syndromes has also revealed that a given phenotype can be caused by multiple genes. A good example of this is with the Rett syndrome, for which the MeCP2 genes is identified in classical patients (4) but for whom, variant cases have been associated with FOXG1 (5, 6), CDKL5 (7). Conversely, a single gene can have multiple phenotypes. For instance, silencing of the Fragile X gene can lead to classical Fragile X syndrome (FXS) or to its Prader–Willi variant, which is characterized by increased appetite and weight gain (8).

**Table 1:** **Common causes of Intellectual disability**.

ID syndrome	ID gene	Prevalence	Clinical phenotype	Heritabilty	*Drosophila* memory
**GENERAL POPULATION**					
Fetal alcohol syndrome	NA	1/200-2000	ID		Not tested
Down syndrome	Down syndrome critical region 1 (DSCR1)	1/650	ID, systemic manifestations	Autosomal/maternal age	Learning+ LTM
Down syndrome	Down syndrome cell adhesion molecule (DSCAM)	1/650	ID, systemic manifestations	Autosomal/maternal age	Learning
Neurofibromatosis	NF1	1/3500	ID, attention difficulty, Tumors	Autosomal dominant/sporadic	Learning+ LTM
Fragile X syndrome	Fmr1	1/4000	Attention difficulty, ID, seizure, autism	X-Linked	Learning+ LTM
Tuberous sclerosis complex	TSC 1 and TSC2	1/12,000	ID, autism, seizures, tumors, systemic manifestations	Autosomal dominant	Not tested
Angelman syndrome	UBE3A	1/15,000	ID, seizures, autism	Autosomal/maternal transmission	LTM
Rett syndrome	MeCP2	1/15,000	ID, regression of skills, microcephaly	X-Linked dominant/sporadic	Not tested
Cri du Chat	CTNND2, TERT	1/20,000	ID, systemic manifestations	90% *de novo*/translocation	Not tested
Williams syndrome	ELN,CLIP2,GTF2I, LIMK1	1/20,000	ID, systemic manifestations	Autosomal dominant/sporadic	Not tested
Prader-Willi syndrome	SNRPN, NDN	1/25,000	ID, Increased appetite	Autosomal/Paternal transmission	Not tested
Rubinstein-Taybi syndrome	CREB-binding Protein	1/100,000	ID	sporadic	Not tested
Mental retardation autosomal recessive 1	Neurotrypsin	Not known	ID	Autosomal recessive	LTM
**SPECIFIC POPULATIONS**					
Tay Sachs	Hexoaminidase A	1/35,000 (jews)	ID, regression	Autosomal recessive	Not tested
Bardet-Biedl	BBS1	1/13,000 (Bedouin)	ID, systemic manifestations, obesity	Autosomal recessive	Not tested
Cockayne	ERCC8	1/300,000	ID, progyria	Autosomal recessive	Not tested
Cornelia De Lange	NIPBL	1/200,000	ID, systemic manifestations	Autosomal dominant/sporadic	Not tested
Phenylketonuria	PAH	1/10,000	ID, systemic manifestations, blond hair	Autosomal recessive	Not tested

*Some of the most commonly seen causes of ID are listed by order of frequency. Some causes are rare in the general population but should be investigated in the high risk specific ethnic groups. Note that the focus is on genetic causes, several other non-genetic causes need to be ruled out and are described in the Section “Memory Formation in Humans and *Drosophila*”*.

The widespread use of genome-wide techniques such as comparative genomic hybridization (CGH) (9) and more recently whole exome sequencing (WES) (10) in patients with ID has allowed for the identification of an increasing number of genetic defects in patients with ID, although in many milder cases a cause can still not be identified (11). Indeed over 528 genes have been found (12) and over 1700 genes are associated with ID in OMIM. A large number of genes have been mapped to the X chromosome [termed MRX and reviewed in Ref. (13)] and account for 5–10% of cases in males (14).

It is important to note that the differential diagnosis for ID also includes cortical malformation, cerebral infections, and ischemic changes that underline the importance of performing MRI in patients with ID. Moreover, ID can also be caused by environmental exposure to drugs, alcohol, or toxins such as lead. In addition, metabolic disorders (see http://www.treatable-id.org/ for a list of disorders) and endocrine dysfunction (such as hypothyroidism) must be ruled out.

Intellectual disability remains without treatment and this can be explained, at least in part, by our lack of understanding of the role of the affected genes in cognitive development. Several animal models have been developed to study the genetic basis of ID, one of which is *Drosophila*. Despite its evolutionary distance to mammals, *Drosophila* has shown positive response to drugs that were later shown to also work in mammals. For instance, metabotropic glutamate inhibitor drugs found to rescue memory in *Drosophila* mutant for the FXS gene (15, 16), the most common single gene cause of ID, also rescues memory in mouse FXS models (17). However, using related compounds in humans failed to show efficacy in recent clinical trials, causing concerns (18, 19). This lack of translation could be caused by several reasons, including the higher level of complexity of the human brain and the increased genetic heterogeneity of humans. Here, we review aspects of *Drosophila* memory studies that may shed light on ID treatment.

First, we will review the basics of learning and memory classification in humans and animal models. Second, we will discuss key cellular mechanisms involved in memory and ID such as the cAMP–CREB pathway, the role of protein synthesis, the function of receptors and anchoring proteins, the role of cell proliferation, and finally the role of neurotransmitters. Third, we will go back to the concept of memory defects in patients with ID and analyze the evidence for such defects in ID patients. Finally, we will discuss key questions relevant to clinical trials for which *Drosophila* learning and memory research may have provided important mechanistic insights.

### Memory formation in humans and *Drosophila*

As mentioned above, the IQ test and ability to learn are at the center of the diagnosis of ID. One of the components of the IQ test is aimed at assessing memory. The study of learning and memory in healthy human subjects was pioneered by Ebbinghaus, who used non-sense word memorization to study memory formation and recall (20). Memory can be classified in several ways: based on temporal pattern, types of information stored, or region of the brain involved in the storage. In the temporal classification, memory can be defined as short-term memory (usually tested a few seconds to a minute after the training) or long-term memory (usually 1 day or 1 week after the training). This type of temporal classification has been widely used in the *Drosophila* study of memory. In humans, short-term memory is also sometimes referred to as working memory. Working memory has been divided conceptually by Baddeley and Hitch (21) into: central executive, a phonological loop, and visuo-spatial sketchpad. Memory can also be classified by the type of memories being stored, implicit or explicit (22). Implicit memory involves the learning of motor skills and depends on basal ganglia and cerebellum. It is spared in hippocampal lesions as shown in the patient HM [reviewed in Ref. (23)]. On the other hand, explicit memory relates to facts (semantic type) or to events (episodic) (24).

Several paradigms have been used to study learning and memory formation in *Drosophila* and include: olfactory, courtship, appetitive, visual, and place memory. Here, because of limited space, we will focus on classical olfactory conditioning, which involves the pairing of an odor with a footshock. Benzer pioneered the assay by training flies using the phototaxis response of flies to induce climbing to a chamber containing an electrified grid where flies would receive a footshock at the same time as an odor was presented (25). Flies would then be allowed to climb to another chamber to receive a second odor but this time without footshock. In 1985, Tully and Quinn modified the assay to allow for the presentation of the odor and the shock in the same chamber without the need for phototaxis (26). This simplified method allowed for higher performance. Using both forward and reverse genetic approaches, several laboratories were able to identify several genes involved in learning and memory that will be described below in Section “Overlap between the Genetics of Memory in *Drosophila* and Intellectual Disability.” As mentioned above, other assays were also developed to take advantage of the power of fly genetics. Indeed, memory of courtship has also been used extensively and relies on the ability of the fly to remember if a female has been courted [reviewed in Ref. (27)]. Appetitive memory is another commonly used paradigm for memory based on positive reinforcement (28).

In addition to identifying genes involved in memory, the assay has allowed the dissection of the persistence of memory as a function of the training provided and the time elapsed since that training. Short-term memory, also known as learning, relates to the memory formed after a single-training session and is tested a few minutes following training. This form of memory is linked with receptor activation and activation of intracellular signaling cascade of secondary messengers but does not require *de novo* transcription (RNA synthesis) or translation (protein synthesis). In *Drosophila*, this memory decays rapidly after 1 day. Intermediate memory has been less well studied and is formed after short-term memory. It depends on the gene amnesia (29). However, some memory can persist up to 1 week, even in flies, and is labeled as long-term memory. Long-term memory is formed after repeated training sessions and is both translation and transcription dependent (30, 31).

Memory studies in *Drosophila* have also shown that different stages of memory depend on various brain regions. The mushroom bodies are required for learning (32). In addition, activity in the mushroom body (MB) innervating neurons (MV1 and MP1 dopaminergic neurons) was shown to be required for long-term memory (33). But more recently, it has been shown that protein synthesis in the dorso-anterior-lateral neurons (DAL) is required for long-term memory formation (34). Memory in flies activates different regions as it goes from short-term memory to long-term memory [reviewed in Ref. (35)]. Several lines of research suggest that information processed by the mushroom bodies could then be stored in the central complex (36). This parallels findings in mammals showing transfer of memory from the hippocampus to the cortex. In humans, information is initially stored in the hippocampus and is later transferred for long-term storage in the cortex for explicit memory and into the cerebellum and basal ganglia for implicit (or procedural memory). This is an important concept in drug discovery and translating any discoveries to other animal models and to human clinical trials because study done in the laboratory in a certain cortical or subcortical area may not translate to other regions or to the global brain function.

### Overlap between the genetics of memory in *Drosophila* and intellectual disability

An important overlap has emerged between genes identified in *Drosophila* memory basic research and genes identified in patients with ID (37). This convergence reinforced the use of the learning and memory models in order to understand the molecular basis of ID. The goal of many reverse genetics studies is to provide insights into the link between gene mutation and cognitive phenotype. We have selected a few pathways to discuss them more in depth, but several other important pathways have been reviewed elsewhere (38).

#### Cyclic Adenosine Monophosphate Signaling Pathway

The cAMP pathway was the initial pathway identified in the forward genetic study of learning in *Drosophila*. The pathway is usually activated via the G-coupled protein. The alpha subunit of the heterotrimetric G protein (Gαs) encodes a GTPase that hydrolyzes GTP to GDP. Mutations in Gαs inhibit the conversion of GTP to GDP, resulting in a constitutively active form of Gαs (39, 40). Gαs modulates cAMP signaling by activating rutabaga, an adenylyl cyclase, which is responsible for cAMP production. Constitutive activation of Gαs in Kenyon cells produces a learning and memory defect in *Drosophila* similar to the ablation of the MB, a key region in memory formation (41).

The *Drosophila* mutant rutabaga (rut) was identified early in the study of *Drosophila* learning and encodes a type 1 Ca^2+^/calmodulin-activated adenylyl cyclase (rut-AC) (42, 43). Rut-AC is a coincidence detector that requires stimulation through G-protein and Ca^2+^/calmodulin to regulate cAMP levels (44, 45). ATP enzymatic conversion to cAMP is mediated through the interaction of the Ca^2+^ binding activity of calmodulin with adenyl cyclase activity that results in an increase in cAMP levels. Rut is required for learning, short-term memory, and long-term memory (46, 47). The degradation of cAMP is performed through phosphodiesterase (PDE). Dunce (dnc) encodes a cAMP-specific phosphodiesterase (dnc-PDE) that is required for learning and leads to the degradation of cAMP (48, 49). Early studies showed that flies with mutation of both dunce and rutabaga combined had significant learning defects (26, 45). Testing of cAMP levels showed that despite showing low levels of both PDE and AC, the dnc rut double mutants had persistently slightly elevated cAMP levels when compared to wild-type flies. Those measurements were made in the abdomen and it remains therefore unknown if brain measurements were also mildly elevated (45). Therefore, antagonizing an enzyme to counterbalance an excess in signaling may not be sufficient in re-establishing normal levels of biomarkers (such as cAMP levels) and may not be sufficient to rescue cognitive defects.

The cAMP-Dependent Protein Kinase (PKA) is a downstream effector of cAMP signaling. An increase in cAMP levels triggers a phosphorylation cascade through the activation of protein kinase A (PKA), and initiates transcription of learning and memory genes (48, 50). Increased levels of cAMP leads to the activation of PKA, DCO is the *Drosophila* PKA catalytic subunit (51). Mutations in the regulatory and catalytic subunits of DCO results in learning and medium-term memory deficits (52–55).

Following activation of PKA or other kinases such as RSK2, the cAMP-Response Element Binding Protein (dCREB2) is phosphorylated, which activates transcription of genes containing a CREB-binding site (56). dCREB2 encodes the transcription factor cAMP response element binding protein (57). dCREB2 is required for long-term memory formation (30, 31, 58). Experimental evidence suggests that CREB protein is required for higher brain functions of MB, which is known to be required for several forms of learning and memory in *Drosophila* and dorsal-anterior-lateral (DAL) neurons. These two regions are joined by synaptic connections, which suggest an interaction between these regions during memory formation and provides a link to the role of CREB in LTM regulation.

Cyclic adenosine monophosphate response element-binding protein’s role in memory is dependent on the nutritional status of the flies though. Hirano found that aversive LTM formation occurred after single-cycle training when mild fasting was applied before training (59). Both fasting-dependent LTM (fLTM) and spaced training–dependent LTM (spLTM) required protein synthesis and cyclic adenosine monophosphate (cAMP) response element-binding protein (CREB) activity. However, spLTM required CREB activity in two neural populations: MB and DAL neurons, whereas fLTM required CREB activity only in MB neurons (60). fLTM uses the CREB coactivator CRTC, whereas spLTM uses the coactivator CBP. In addition to the change in co-factor requirement, starvation was shown to change neuronal activity in dopaminergic neurons activated by memory training (60).

Several ID genes have been shown to affect the cAMP–CREB pathway. An important ID gene linked to *Drosophila* memory was the human CREB-binding protein (CBP or CREBBP). As mentioned above, CREB requires the co-factor CBP. CBP has been found to be mutated in patients with Rubinstein–Taybi syndrome (RTS) (MIM180849) (61), a rare cause of syndromic ID. Drug rescue with PDE inhibitors was shown in the mouse model of RTS (62).

Another important ID gene linked to the cAMP pathway is Neurofibromin (NF1) (MIM 613113), which encodes a Ras-specific GTPase-activating protein (GAP) (63). In neurofibromatosis type 1 (NF1), the NF1 gene contains mutations that alter functioning of this protein. NF1 is a neurocutaneous syndrome in which patients have skin and brain symptoms and signs. Patients with NF1 suffer from attention difficulty, learning disabilities, and intellectual disabilities. A *Drosophila* homolog of NF1 is expressed broadly in the adult fly brain including key structures associated with learning and memory such as the MB, lateral horn, and antennal lobes (64). NF1 regulates adenylyl cyclase activity (rut-AC) and cAMP production (41, 63, 65–68). NF1 acts as a GAP on Gαs thereby altering rut-AC dependent synthesis of cAMP. NF1 is required for memory acquisition and short-term memory but interestingly, different domains are required for each phase of memory (69).

Finally, the ID gene Fragile X mental retardation 1 (FMR1) (OMIM 309550) has also been shown to affect the cAMP pathway. FMR1 is mutated in patients with FXS, the most common cause of ID in boys. Berry-Kravis showed that cAMP levels in response to activation (with forskalin) were lower in patients with FXS (70). *Drosophila* has a highly conserved homolog of FMR1 (71), dfmr1. Dfmr1 regulates local mRNA translation in dendrites (72, 73). *Drosophila* mutant for dfmr1 has been shown to have courtship and olfactory learning and memory defects. Recently, FX memory defects in *Drosophila* were shown to be reversed by treatment with PDE inhibitor (74, 75). Moreover, CREB level circadian fluctuations have been shown to be defective in FX-mutant flies. In addition, higher levels of FMRP are seen in PDE mutants (15, 74).

#### Protein Synthesis and Degradation

**Figure 2:**
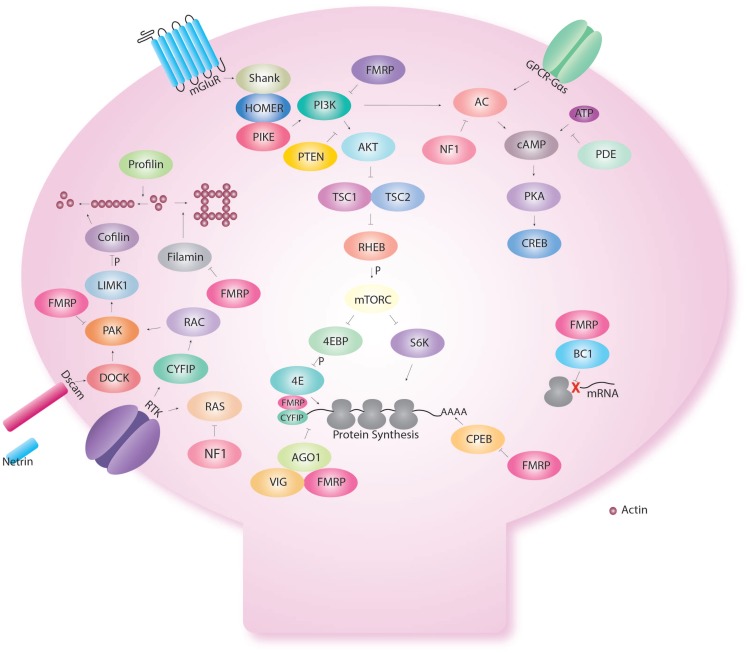
**Fragile X Mental retardation protein regulates several cellular functions in the dendritic spine**. Fragile X Mental retardation protein affects multiple aspects of neuronal metabolism. Even within a certain function like translational control, FMRP interacts with multiple pathways. Indeed, FMRP has been shown to regulate the AKT pathway via repression of PIKE. In addition, FMRP regulates CPEB that regulates translation via the poly A tail. FMRP also binds CYFIP and is part of the microRNA and short interfering pathway. FMRP also regulates the assembly of the ribosome to target RNA with the short RNA BC1. FMRP also interact with the cAMP–CREB pathway via the link with PKA but also because FMRP is produce in response to CREB activation. FMRP regulates cellular shape via actin remodeling using multiple molecules (profilin, cofilin, filamin) and receptors (Dscam, Receptor tyrosine kinase-RTK).

Interestingly though, FMR1 is not only involved in cAMP signaling but is also involved in control of protein synthesis (Figure 2). Early biochemical studies showed a global up-regulation of protein synthesis in FX (76–80). FMRP is an RNA binding protein for which several targets have been identified (77, 81–83). We showed that excess protein synthesis was deleterious to memory in *Drosophila* but could be reversed with the protein synthesis inhibitors, cycloheximide and puromycin (15). FMRP has been shown to regulate protein synthesis via the AKT pathway, microRNA (15), and CPEB (84). Indeed, Sharma et al. showed that AKT signaling was enhanced in the mouse model of FX (85). This was followed by the demonstration of excess AKT signaling in the *Drosophila* larva brain (86). Loss of FMRP results in the over-activation of mGluRs (74), which will be discussed in the next section on receptors.

The PI3K–mTOR pathway includes other ID genes: TSC1 and TSC2. The *Drosophila* Tuberous sclerosis (dTsc1 and dTsc2) is homologs of TSC1 and TSC2, which are mutated in patients with Tuberous Sclerosis complex (OMIM 191100; OMIM 191092). Patients affected by TSC have brain, skin, heart, kidney, eyes, and lung defects. Cognitive symptoms will include ID and autism. dTsc1 and dTsc2 function downstream of PI3K/AKT. dTsc1 and dTsc2 form a heterodimeric complex in which dTsc2 is the catalytic subunit containing a GAP domain for RHEB (87). Overexpression of RHEB in *Drosophila* resulted in defect in 3-h appetitive memory leaving the immediate memory intact (88). Mutations in TSC genes lead to excess protein synthesis (similar to FX) (89–91). Downstream of TSC genes is the Mammalian Target of Rapamycin (mTOR). mTOR is a serine/threonine kinase that belongs to the phosphatidylinositol 3-kinase-related protein family. mTOR functions as part of two distinct signaling complexes, mTORC1 and mTORC2. Both mTORC1 and mTORC2 function at synapses to regulate mRNA translation (92–94). mTORC1 is activated through the direct binding of RHEB. mTORC1 targets and phosphorylates eukaryotic initiation factor 4E binding proteins (4EBPs) and ribosomal protein kinases (S6Ks) (89). S6K in turn targets other translation regulators (95). Inhibiting the function of mTORC1 creates defects in long-term memory formation (96, 97). mTORC2 can be directly activated through the PI3K pathway via phosphatidylinositol (3,4,5)-trisphosphate (PIP3) (98). mTORC2 activity is elevated during stimuli-associated learning and has recently been identified to have a role in mGluR dependent long-term depression (94). Recently, it was shown that removal of the downstream target S6K in FX mutant mice rescues behavioral defects including memory, reinforcing the role of protein synthesis in FX (99).

A novel gene involved in the AKT pathway is Arouser (aru). Aru has been implicated in short-term memory deficits in *Drosophila* in a background-specific manner (100). Although not fully characterized in *Drosophila*, aru is homologous to mammalian to the Epidermal growth factor receptor kinase substrate 8 (EPS8) protein family, Eps8L3 (101). Eps8L3 is an actin-capping protein that functions in the epidermal growth factor receptor signaling pathway (Egfr) as well as the PI3K/AKT signaling pathway and is needed for normal spine morphology, synaptic plasticity, and memory formation (102). Abnormal spine morphology has been associated with ID and autism (103, 104). Esp8 knockout mice exhibit learning and memory impairment (105). Furthermore Eps8 levels are decreased in patients with autism (105).

**Figure 3:**
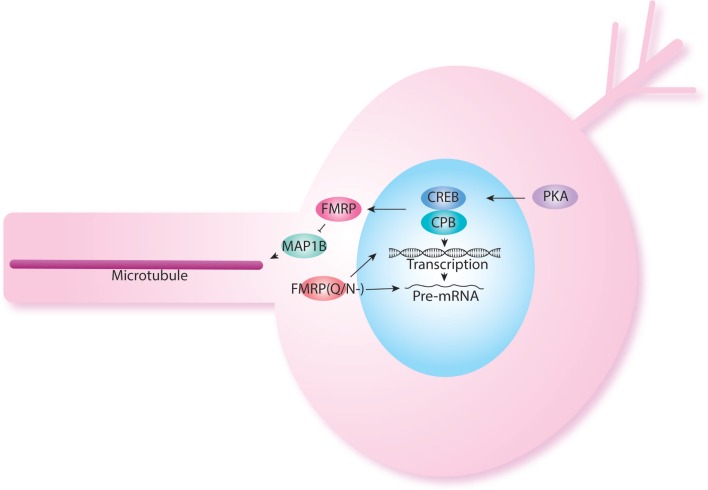
**FMRP also acts at the nuclear level**. FMRP has been shown to be produced in response to cAMP–CREB activation. Indeed, levels of FMRP are increased in PDE mutants. FMRP isoforms have been shown to have different role in short-term and long-term memory as was shown previously in NF1. The FMRP isoform lacking the Q/N rich domain is localized back to the nucleus and may participate in histone modification and splicing.

Another important regulator of protein synthesis is the cytoplasmic polyadenylation element binding protein (CPEB). oo18 RNA-binding protein 2 (Orb2) is an important member of the CPEB family. Orb2 regulates local mRNA translation at pre- and post-synaptic compartments (106–110). In *Drosophila*, Orb2 is specifically required in MB γ neurons for long-term memory formation (111). Orb2 encodes two isoforms, Orb2A and Orb2B (111). Orb2A and Orb2B differ in their N-terminal domains, but have similar C-terminal domains. Together Orb2A and Orb2B form a heterodimeric complex, this interaction is mediated through the Q-domain of Orb2A that function at synapses (112). The Q-domain of Orb2A is specifically required for long-term memory but not short-term memory (112). FMRP has been shown also to interact with Orb proteins in protein translation regulation (84). Interestingly, specific FMRP isoforms without the glutamine/asparagine (Q/N-) are required for LTM when most isoforms, which contain Q/N rich sequence in the C-terminal region of the protein are sufficient for STM (113). The Q/N-isoform shows a predominantly nuclear localization and has been hypothesized to relate to regulation of chromatin and mRNA splicing and transport (114) (Figure 3). The interaction between FMRP and CPEB has been also confirmed in the Richter lab who showed that decreasing CPEB activity in mouse could rescue memory defects seen in FX mice (115).

Protein levels are also regulated through their degradation. Ubiquitin-dependent proteasome degradation is an important pathway for protein degradation. Mutation in the maternally inherited copy of the ubiquitin ligase UBE3A have been identified in 65–75% of Angelman syndrome (OMIM 105830) cases (116). The remainder of cases is linked to paternal uniparental disomy, imprinting defects, and UBE3A mutations (117). The *Drosophila* model of Dube3A has shown that Dube3A is required for synaptic potentiation and long-term synaptic depression (118, 119) and that disruption of Dube3A leads to robust behavioral defects, including deficits in climbing activity, circadian rhythms, and long-term associative olfactory memory (120).

The serine-protease neurotrypsin was also shown to be important in cognition in both human and flies (121). Indeed, humans with neurotrypsin mutations suffered from autosomal recessive ID whereas *Drosophila* showed long-term memory specific defects.

#### Receptors, Anchoring Proteins, and Neurotransmitters

*Drosophila* group II metabotropic glutamate receptor gene (DmGluRA) has gathered a lot of interest with its implication in FXS (122–124). DmGluRAs are G-protein-coupled receptors that are present in MB calyces (125, 126). The binding of glutamate to the receptor results in the down-regulation of neuronal activity via AMPA receptors internalization in a protein synthesis/PI3K-dependent manner (127).

Indeed, Huber showed that the enhanced long-term depression in FX hippocampus was triggered by excessive mGluR activity. This is because FMRP regulates the protein synthesis-dependent internalization of AMPA receptors in addition to the levels of mGluR. The net result of the excess protein synthesis in absence of FMRP was enhanced internalization of AMPA receptors. Following that, mGluR inhibition in *Drosophila* FX mutants was shown to rescue memory defects (15, 16). There is also a feedback loop wherein activation of mGluRs usually up-regulates FMRP through the cAMP-CREB pathway (128, 129).

*Drosophila* NMDA receptors 1 and 2 (dNR1 and dNR2) encode NMDAR receptors that regulates excitatory neurotransmission via glutamate (130). Synaptic changes occur following the binding of glutamate to the receptor and the subsequent membrane depolarization that results in Ca^2+^ influx into post-synaptic cells (131). Mutations and disruptions to the expression of dNR1 impair learning and long-term memory (130, 132). Recently, anti-NMDA receptors antibodies encephalitis was shown to be the cause of autism-like symptoms (133).

Down syndrome (MIM 190685) represents the most common cause of ID and is caused by trisomy of chromosome 21. It causes a syndromic ID associated with progressive dementia as well as a host of other systemic manifestation. A critical region has been recognized within chromosome 21. Nebula (nla) the *Drosophila* homolog of Down syndrome critical region 1 gene (DSCR1) encodes a calcipressin that binds to and inhibits calcineurin, and is required for learning and long-term memory (134). Calcineurin 1 is regulated by nla by inhibiting calcineurin-mediated signaling (135). Altered nla expression results in learning and memory defects. Calcineurin targets and up-regulates the activity of synaptojanin (synj) via its dephosphorylation (136). Synj encodes a phosphatidylinositol phosphatase that functions in endocytosis (137). Minbrain (Mnb), which encodes a serine/threonine kinase, targets and phosphorylates synj (138). Synj and nla are overexpressed in DS (139).

#### Neuronal Proliferation

The Sry-related high mobility groove box genes (SOX) in human have important roles in neuronal proliferation and early development. Mutation in SOX3 has shown to be causative in non-syndromic ID (140). Dichaete is a member of SOX gene family in *Drosophila*. This gene is expressed in olfactory bulb neural precursors and granule cells and was found to have an influence on wide range of developmental processes. Dichaete functions in the development of the CNS by regulating numerous genes (141–143). In the adult fly brain, dichaete is expressed in glia and neurons of the olfactory circuit but there is no data on memory formation at this time (144).

#### Cytoskeleton, Anchoring Protein, and Cell Adhesion

Receptors need to be properly localized to the synapse to perform their function. Several anchoring proteins genes have been found in patients with ID. Recently, the postsynaptic domain (PSD) protein SHANK2 gene was identified in ID and ASD cases through microarrays (145). SHANK2 (also known as prosap) type of molecules links the actin cytoskeleton to the surface receptors such as NMDA and mGluR. Thus far only change in alcohol sensitivity has been shown in the *Drosophila* prosap mutants (146). It may be interesting to test learning and memory as several memory mutants had abnormal alcohol sensitivity (147). Mutations in SHANK3 have also been identified in ID patients. These mutations lead to loss of binding of another PSD protein, Homer (148). Homer-related proteins are part of large complex responsible for structural and functional plasticity of glutamatergic synapses. In *Drosophila*, a Homer homolog was identified and found to be expressed in the nervous system (149). Moreover, courtship memory was defective in *Drosophila* homer mutants (149).

Dscam encodes a protein kinase that is required for segregation of sister branches of the axons of MB neurons (150, 151). It functions by binding extracellular molecules like netrin and then transducing the signal from the extra to intra cellular milieu through Dock and PAK resulting in change in the actin cytoskeleton. Dscam is translated locally in dendrites and likely contributes to synaptic plasticity (152). Dscam is regulated by FMRP. FMRP null mutants have increased Dscam levels, which results in abnormal behavioral responses and increased error in synaptic targeting (153, 154). Taken together, dscam provides evidence of a possible link between DS and FXS (154).

Dfmr1 has also been shown to interact with another ID gene, Filamin. Some patients with FX presented with periventricular nodular heterotopia (PNH), a neuronal migration defect associated with learning difficulties and seizures (155). PNH was previously linked to mutation in Filamin. *Drosophila* mutant for Filamin homolog, cheerio, were found to have long-term memory defects. Interestingly, cheerio and dfmr1 interact together in formation of olfactory long-term memory (156).

### Learning and memory defects in intellectual disability

Despite the extensive use of behavioral memory assays and electrophysiological models (such as long-term potentiation and depression) in model organisms of ID genes, there is still significant gap in our understanding of memory defects in ID. This is particularly a problem for non-verbal patients. Moreover, co-morbid problem such as autism and attention deficit have made memory testing in ID patients more challenging. Here, we will review the known defects of memory, mostly short-term memory, in patients with ID.

**Figure 4:**
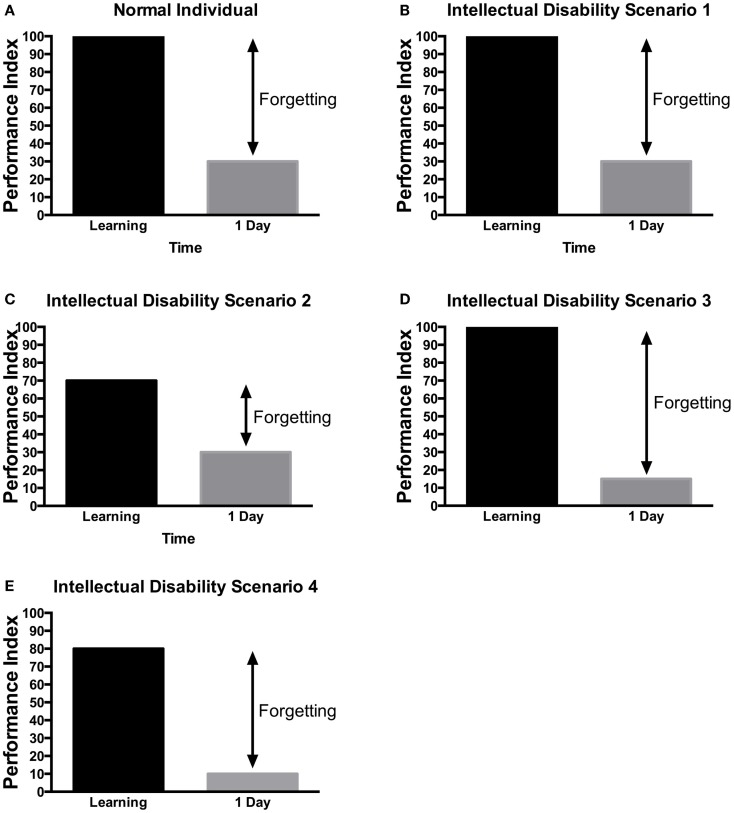
**Scenarios of learning and memory defects in patients with ID**. Several potential scenarios could be proposed to account for cognitive defects of patients with ID based on previous research findings. Depending on the gene tested or the method used, these various scenarios have been observed in ID patients. **(A)** In control, subjects without ID will be able to learn a task to criterion and then will present a lower performance in retest at 24 h. **(B)** In some rare cases, no learning and memory defects would be observed. **(C)** In some patients, defect in learning could be the sole manifestation. **(D)** In other patients, they can learn the information but would not be able to retain it. **(E)** Finally, in some patients, learning and long-term memory could be both impaired. The decreased level of learning, could be responsible for the defect in memory as the actual forgetting levels are the same as in controls. It should be noted that at this time, there is little systematic study aimed at identifying which model is most applicable to each specific genes. These hypothetical scenarios may provide a framework for the memory investigation in ID.

As mentioned earlier, the work of Ebbinghaus showed that the average recall of learning information would be around 30% of the information learned (20). Several conceptual hypotheses have been suggested for learning and memory in ID patients (Figure 4). One possibility is that patients with ID have no learning and memory defect (Figure 4B) but rather other general mental ability defects that limit their cognitive performance. This has been mostly seen in patients with severe learning disability (157). Alternatively, they may have defect of acquisition (Figures 4C,E), consolidation (Figure 4D), recall or long-term memory in isolation. As ID is defined by defect in IQ and adaptive functions, children may perform the Weschler Intelligence Scale, which includes a test of digit span memory. This test is given to children aged 7 and above. It requires the child to repeat the digit named either forward or backward. In many children with ID, the task may be too difficult to perform. Nonetheless, various studies have aimed at defining potential memory defects in ID.

Early studies focusing on short-term memory showed defects in memory in patients with ID when compared to age-matched subjects (158, 159). Long-term memory studies, when matched for mental age showed mixed results [reviewed in Ref. (160)]. Some studies found that the defects in ID patients were the consequence of initial encoding defects (159, 161) with the same rate of forgetting as in control individuals (as in Figure 4E). On the other hand, others studies showed that the memory decay was increased in patients with ID (as in Figure 4D). For instance, Katz (162) showed that individuals with mild and moderate ID had defects in remembering items (naming of pictures) immediately when compared to college students. When tested at 24 h, ID patients presented a bigger loss in memory than the college students did. It is interesting that the college students exhibited little to no forgetting. This may raise questions about the level of difficulty of the task and the presence of reconsolidation in the student group as opposed to the ID patients. In the same study, memory for the location of object was found to be defective only in patients with moderate ID. Instruction at the time of encoding affected the level of memory impairment in patients with ID. These studies frequently enrolled patients based on the IQ without knowledge of the etiology of their ID. This may pool together patients with different learning and memory pattern and confound the studies.

More recently, studies of memory have been targeting specific genetic etiology. Studies of Down syndrome patients revealed that explicit memory was more severely affected in patients with ID (163). This was noted to be true for short and long-term memory in explicit memory. Tasks used in these studies included word lists, word completion, and prose recall. In the same study, difference in performance between DS and ID patients emerged in learning tasks. For instance, DS patients learned similarly words that were related or not, but other ID patients did better when learning words that were related. Also in that study, assessment of the long-term memory showed that the decay of memory was similar between normal subjects, patients with Down syndrome, and other patients with ID (as in Figure 4E). In addition, Carlesimo (163) showed that implicit memory was actually normal in individuals with ID (as in Figure 4B).

Selective long-term memory deficit was observed by Vicari (164) in patients with Williams syndrome (as in Figure 4C). Patients had to copy Rey Figures at various time points and the number of items remembered in the figures was scored. Vicari showed impaired long-term memory due to increased forgetting rate in patients with Williams syndrome (164).

In contrast to the limited number of studies on long-term memory, there is a large amount of studies examining working memory. As noted above, several studies have suggested that the defect in early stage of encoding and attention are the cause for poor long-term performance in ID (165). Baddeley’s model of working memory includes (1) a central executive component, (2) a phonological loop, and (3) a visuo-spatial sketchpad (166–168). Each component can be tested separately in patients. Various degrees of working memory defects have been observed in children with ID (169–171). Defects in working memory were observed in children with learning disability even in presence of normal IQ, which raises the need to distinguish ID from LD in memory studies (172). Moreover, Henry showed that the level of performance in the phonological and visual spheres was matched to the mental age and did not show a specific pattern (173). The mean non-verbal IQ was 60 and there were no patients with Down syndrome in the cohort.

Again, etiology specific defects have been observed. For instance, Down syndrome patients were found to have defects in verbal and spatial backward spans (remembering a string of object but in a reverse manner) aspects of working memory (174). The IQ in that study was averaged at 36. The patients with DS had performance similar to ID and mental age-matched subjects when the task involved remembering the objects in a forward manner.

Another ID population studied extensively is the FX. Short-term visual and verbal memory defects were identified early on (175). Working memory defects were shown to be present only when tasks were complex (176). A signature pattern of defects in attention, impulsivity and working memory was observed by several groups [reviewed in Ref. (177)]. FX patients have defects in several aspect of working memory: auditory, visuospatial, and memory for words (178). In the same study, FX patients with ID were more severely affected.

### Insight from flies learning and memory for drug development in ID

Reports from recent large randomized controlled trials failure questioned the value of pre-clinical research in animal models (19). There have been important findings from *Drosophila* memory research, which could provide insight into how to successfully translate candidate drugs from the lab to the clinic. Here, we provide a brief discussion highlighting some factors that need to be taken into account when thinking about applying basic science findings in flies to clinical trials. We also speculate on some other potential pitfalls raised in basic science research that have not yet been formally tested in ID research.

#### Protein Domain-Specific Effects on Stage of Memory Formation

In clinical studies or drug trials, patients are grouped based on their diagnosis (FXS, NF, etc.). But as shown in the study of learning and memory in the *Drosophila* mutants of FXS (113) and Nf1 (69), different regions of the protein can either affect learning and/or memory. This may need to be considered in the selection of the patients for clinical trials as a drug aimed at learning may not have an effect for the patients in whom the mutation affects solely memory (Figure 4). In addition, cognitive testing may need to test attention skills and different phases of memory (working memory and long-term memory) to assess for inter-patients variability, more homogenous baseline values, and responses to drugs.

#### Translating Outcome from Homogenized Backgrounds to Real Populations

Many drugs are studied *in vitro* and then in standardized assay with animal on a homogenous genetic background. The phenomena of genetic background-specific gene expression can also provide an explanation for varying degrees of clinical phenotypes (179). In *Drosophila*, MB morphology differs when MB miniature (mbm) is placed into different genetic backgrounds (180). In addition, recent data in the arouser (aru) mutant has shown that the memory defects were variable in function of the background (100).

#### The Effect of Nutritional Status on Gene Expression

This concept has emerged recently, thanks to two studies published last year by the Saitoe (59) and Preat (60) laboratories. They showed that molecular co-factor of CREB would differ depending on the fact that the flies were starved prior to long-term memory training or not. In addition, neuronal activity in key dopaminergic neurons was altered by starvation. This is important when looking at children with ID. Indeed, several children will have feeding abnormalities, with either lack or atypical feeding due to sensory-processing issues or excess feeding as part of obsessive-compulsive behaviors (181). This may affect the metabolic state of the patients and then impact their response to drugs.

Other factors have not been as well documented in *Drosophila* but have been suggested from *Drosophila* research.

#### Neuronal Network

It is important to consider that information transits from one brain part to another as memory is learned, consolidated, re-consolidated, and then recalled. Moreover, the time elapse since acquisition (short-term versus long-term) may influence in which part of the brain the memory is located. This has been extensively studied in *Drosophila* (32, 182–184). It is therefore important to characterize the type/stage of memory in animal models and ID patients when considering transposing a candidate drug. The increased network complexity in humans CNS (considering for instance the expansion of the frontal lobe compared to mouse or rats) may lead to differential reactions between animal models and human.

#### Genes can Affect Multiple Pathways

This is well recognized in cancer treatment and infectious resistance. However, this is still poorly understood in ID. Homeostasis through parallel pathways and receptor turnover can compensate for modification made with a drug. A good example of a single gene having effect through multiple pathways is FX (Figures 2 and 3) in which multiple pathways interacting are regulated by FMRP. This may give raise to phenotypic overlap between syndromes. In addition, it may explain the phenotypic variation within a given syndrome depending on the burden put on each pathway by other modifier genes present in an ID patient.

#### Environmental Effect on Performance

Probably one of the most important issues with animal models is the fact that conditions are well controlled. Indeed, scientists studying animal behavior will tell you that rearing, environmental, and experimental context conditions need to be extremely well controlled in order to provide successful and reproducible experiments. This “perfect day” scenario is hard to obtain in the clinical trials, especially with ID children that frequently have sensory-processing issues, high level of anxiety in novel or test situations, and obsessive behaviors. Indeed, the comorbidity observed in FXS patients for instance [reviewed in Ref. (185)] (Figure 1) are frequently observed even in *Drosophila* and are carefully controlled in experimental manipulations. These include anxiety (186), sleep problems (187), and social interaction defects (188). Considering that anxiety can have a significant and fluctuating effect on ID patient’s behavior and memory (189), this should be monitored more closely in trials.

## Conclusion

Increasingly sensitive genome-wide techniques have provided researchers and clinicians with an increasing yield of identifying specific etiologies in patients with ID (12). This has led to a larger list of genes involved in ID. Nonetheless, mechanistic insights and treatments have lagged behind. This gap may become larger if ID genes to be discovered are of low prevalence and/or small effect. The memory assays in *Drosophila* have emerged as important model to study the molecular basis of cognitive defects linked to ID genes. *Drosophila* can also be used to screen pharmacological compounds for their ability to rescue memory defects observed in ID model flies. Several pitfalls need to be monitored in order to increase the yield of translating findings from the lab into clinical successes.

## Author Contributions

AA, BA-J, and FB performed the literature research and wrote the paper; AA and FB designed the figures.

## Conflict of Interest Statement

The authors declare that the research was conducted in the absence of any commercial or financial relationships that could be construed as a potential conflict of interest.

## Abbreviations

4EBP, eukaryotic translation initiation factor 4E-binding protein 1; AKT, serine/threonine-specific protein kinase; AMPA, α-amino-3-hydroxy-5-methyl-4-isoxazolepropionic acid; aru, arouser; ASD, autism spectrum disorders; ATP, adenosine triphosphate; cAMP, cyclic adenosine 3′,5′-monophosphate; CBP/CREBBP, CREB-binding protein; CGH, comparative genomic hybridization; CNS, central nervous system; CPEB, cytoplasmic polyadenylation element binding protein; CREB, cAMP response element binding protein; CRTC, CREB regulated transcription coactivator 1; DAL, dorso-anterior-lateral neurons; DCO, *Drosophila* PKA catalytic subunit; dCREB, *Drosophila* cAMP-response element binding protein; dfmr1, *Drosophila* fragile X mental retardation 1; DmGluRA, *drosophila* group II metabotropic glutamate receptor gene; dnc, dunce; dnc-PDE, dunce encoded cAMP-specific phosphodiesterase; dNR1, defense repressor 1; dNR2, defense repressor 2; DS, Down syndrome; Dscam, Down syndrome cell adhesion molecule; DSCR1, Down syndrome critical region 1 gene; dTSC1, *Drosophila* tuberous sclerosis complex 1; dTSC2, *Drosophila* tuberous sclerosis complex 2; Egfr, epidermal growth factor receptor signaling pathway; EPS8/Esp8, epidermal growth factor receptor kinase substrate 8; fLTM, fasting-dependent long-term memory; FMR1, fragile X mental retardation 1; FMRP, fragile X mental retardation protein; FOXG1, forkhead box protein G1; FX, fragile X; FXS, fragile X Syndrome; Gαs, alpha subunit of the heterotrimetric G protein; GDP, guanosine diphosphate; GTP, guanosine-5′-triphosphate; ID, intellectual disability; IQ, intellectual quotient; LD, learning disability; LTM, long-term memory; MB, mushroom body; MeCP2, methyl-CpG-binding protein 2; mGluR, metabotropic glutamate receptors; Mnb, minbrain; MP1, mushroom body innervating dopaminergic neuron P1 cluster in protocerebrum; MRI, magnetic resonance imaging; mRNA, messenger ribonucleic acid; MRX, mental retardation X linked; mTOR, mammalian target of rapamycin; mTORC1, mammalian target of rapamycin complex 2; mTORC2, mammalian target of rapamycin complex 1; MV1, mushroom body innervating dopaminergic neuron V1 cluster in protocerebrum; Nf1/NF1, neurofibromatosis type 1; nla, nebula; NMDAR, *N*-methyl-d-aspartate receptor; Orb2, oo18 RNA-binding protein 2; PDE, phosphodiesterase; PI3K, phosphatidylinositol (3,4,5)-trisphosphate; PIP3, phosphatidylinositol 3,4,5 trisphosphate; PKA, protein Kinase A; PNH, periventricular nodular heterotopia; PSD, postsynaptic domain; Q/N, glutamine/asparagine; RHEB, RAS homolog enriched in brain; rut, rutabaga; Rut-AC, rutabaga encoded type 1 Ca^2+/^calmodulin-activated adenylyl cyclase; RNA, ribonucleic acid; RSK2, ribosomal S6 kinase; RTS, rubinstein-taybi syndrome; S6K, ribosomal protein S6 kinase; SHANK2, SH3 and multiple ankyrin repeat domains protein 2; SHANK3, SH3 and multiple ankyrin repeat domains 3; SOX, SRY-related high mobility groove box; SOX3, SRY (sex determining region Y)-box 3; spLTM, spaced training long-term memory; STM, short-term memory; synj, synaptojanin; TSC1, tuberous sclerosis complex 1; TSC2, tuberous sclerosis complex 2; UBE3A, ubiquitin protein ligase E3A; WES, whole exome sequencing.
